# Supplementary material for: Methylphosphonate-driven methane formation and its link to primary production in the oligotrophic North Atlantic
Source: Nat Commun. 2023 Oct 16;14:6529. doi: 10.1038/s41467-023-42304-4 (PMC10579326; doi:10.1038/s41467-023-42304-4)
Supplement: Supplementary file 1 — Supplementary Information [file 41467_2023_42304_MOESM1_ESM.pdf]

Supplement

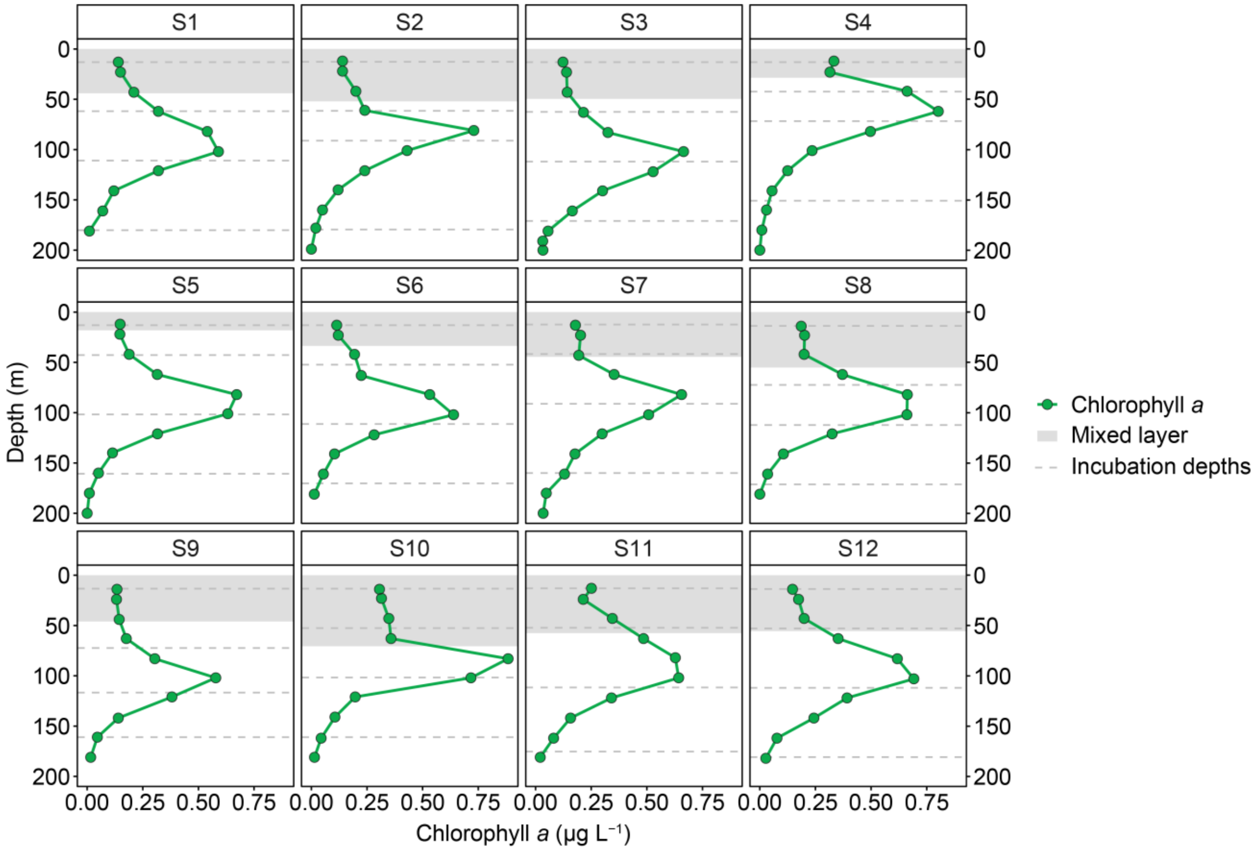

Figure S1. **Surface mixed layer in each station in relation to the deep chlorophyll maximum.** Mixed layer was defined as depth at which potential density is by 0.3 kg m^{-3} larger than at reference depth at 10 m. The surface depth was always located within the mixed layer, but the intermediate depth was only in the mixed layer for stations S7, S10, S11 and S12. The deep chlorophyll maximum was consistently below the mixed layer. Source data are provided as a source data file.

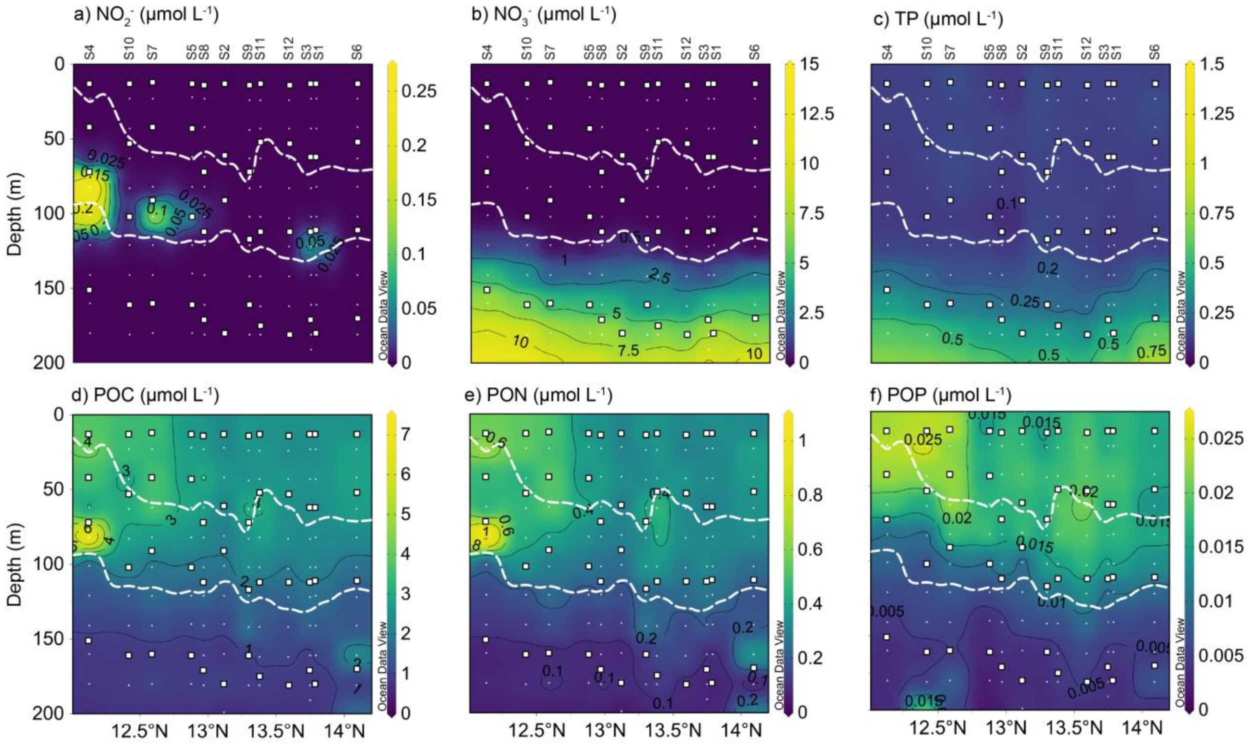

Figure S2. **Additional chemical parameters of the upper 200 m of the water column in the western Tropical North Atlantic east of Barbados. a,** Nitrite (NO_2^-). **b,** Nitrate (NO_3^-). **c,** Total phosphorus (TP). **d,** Particulate organic carbon (POC). **e,** Particulate organic nitrogen (PON). **f,** Particulate organic phosphorus (POP). White squares represent depths of the incubation experiments and white circles indicate additional sampling depths for concentration measurements. The white dotted line represents the outline of the deep chlorophyll maximum, defined as the area between 0.35 µg L^{-1} chlorophyll *a*. Source data are provided as a source data file.

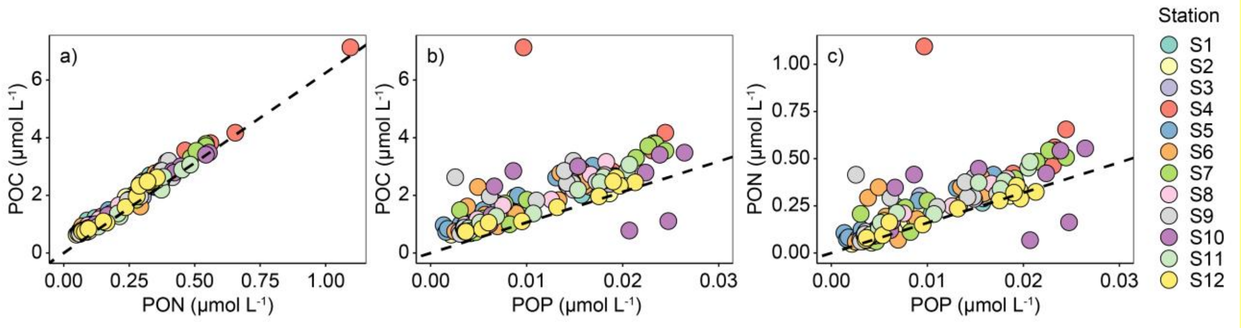

Figure S3. **Carbon, nitrogen and phosphorus ratios in the particulate organic fractions. a,** POC:PON. **b,** POC:POP. **c,** PON:POP. The lines represent the Redfield ratio of 106:16:1 (C:N:P). Source data are provided as a source data file.

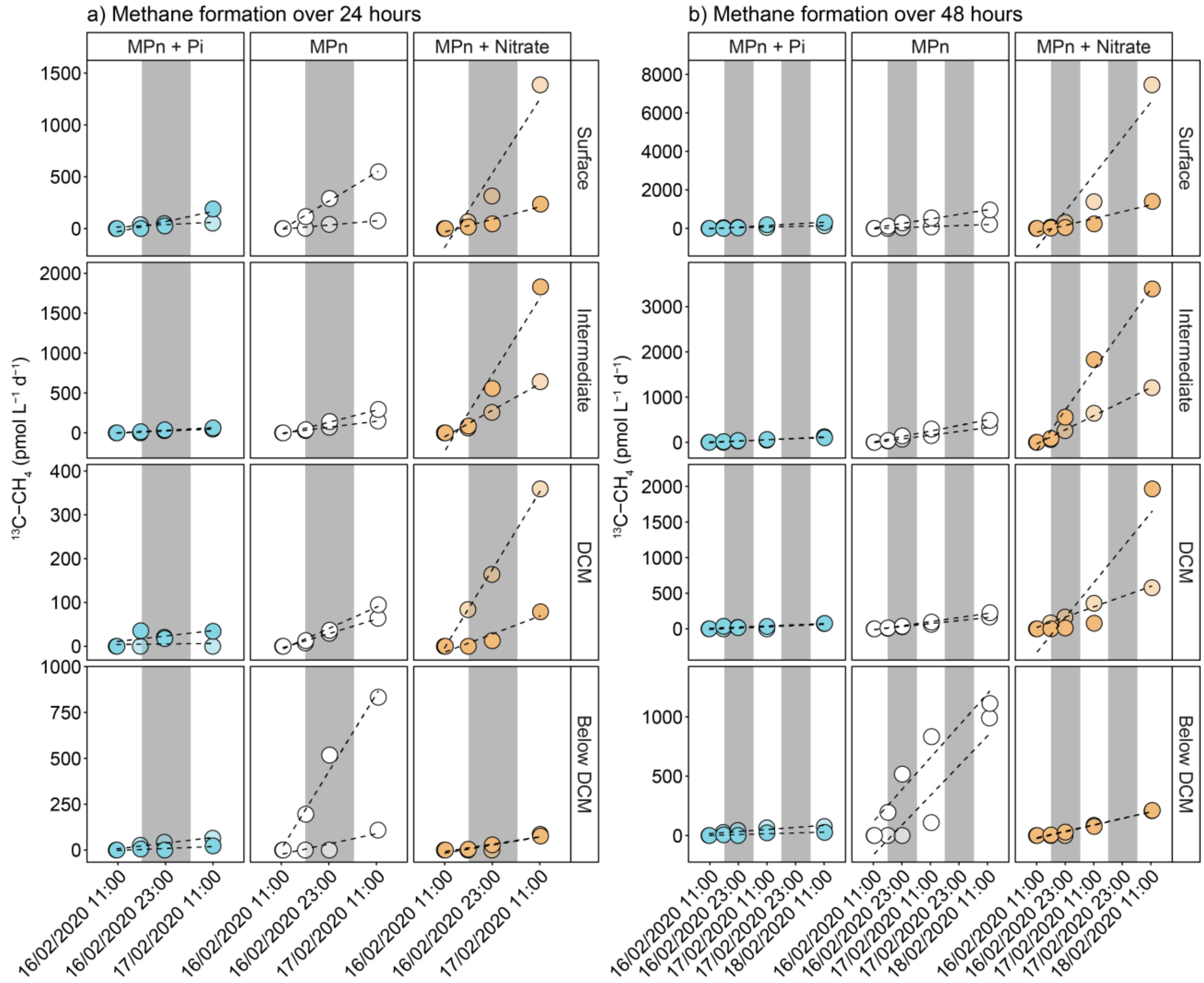

Figure S4. **Methane formation from methylphosphonate over time in representative incubations from station 11. a,** Methane formation over 24 hours of incubation. Rates were tested for linearity and significance prior to rate calculation. **b,** Methane formation over the complete 48 hour incubation period. Rate measurements were from the four incubation depths: 10-metre surface depth, intermediate depth, deep chlorophyll maximum (DCM) and below the DCM and the three incubation experiments: methylphosphonate-addition (MPn; white), methylphosphonate + phosphate-addition (MPn + Pi; blue) and methylphosphonate + nitrate-addition (MPn + nitrate; orange). While some rates remained linear over this time period, often exponential rates could be observed. Replicate incubations are shown. Dark bars represent the time between sunset and sunrise. Source data are provided as a source data file.

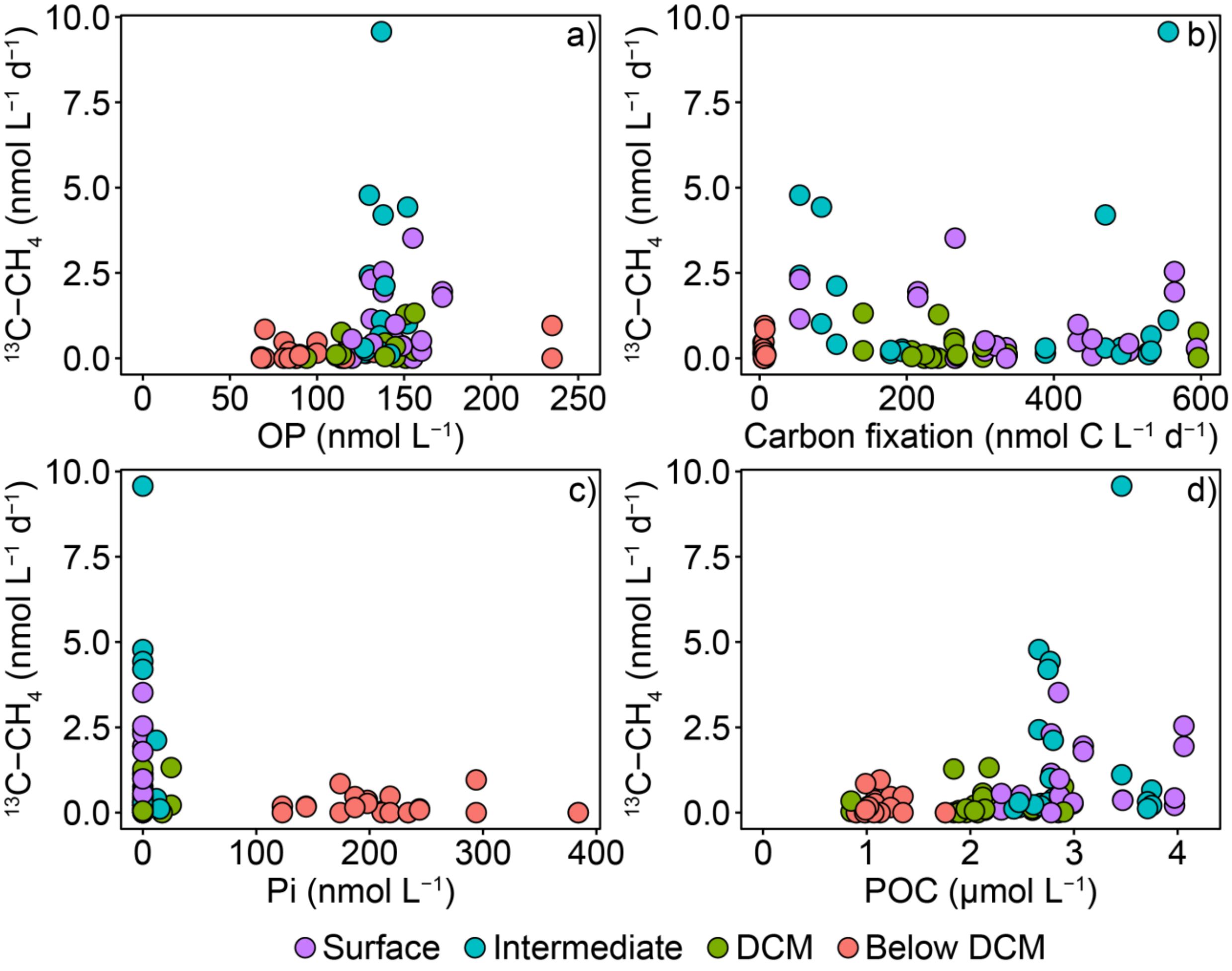

Figure S5. **Methane formation from the methylphosphonate-addition experiment as a function of organic phosphorus (OP) concentrations (a), carbon fixation rates (b), phosphate (Pi) concentrations (c) and particulate organic carbon concentrations (d).** The depths from all twelve stations were used: a 10-metre surface depth, intermediate depth, deep chlorophyll maximum (DCM) and below the DCM, except for S2 where nutrient data was unavailable. The mean rate of the triplicate carbon fixation incubations is presented in the graph, whereas the duplicates of the methylphosphonate incubation are shown separately. Source data are provided as a source data file.

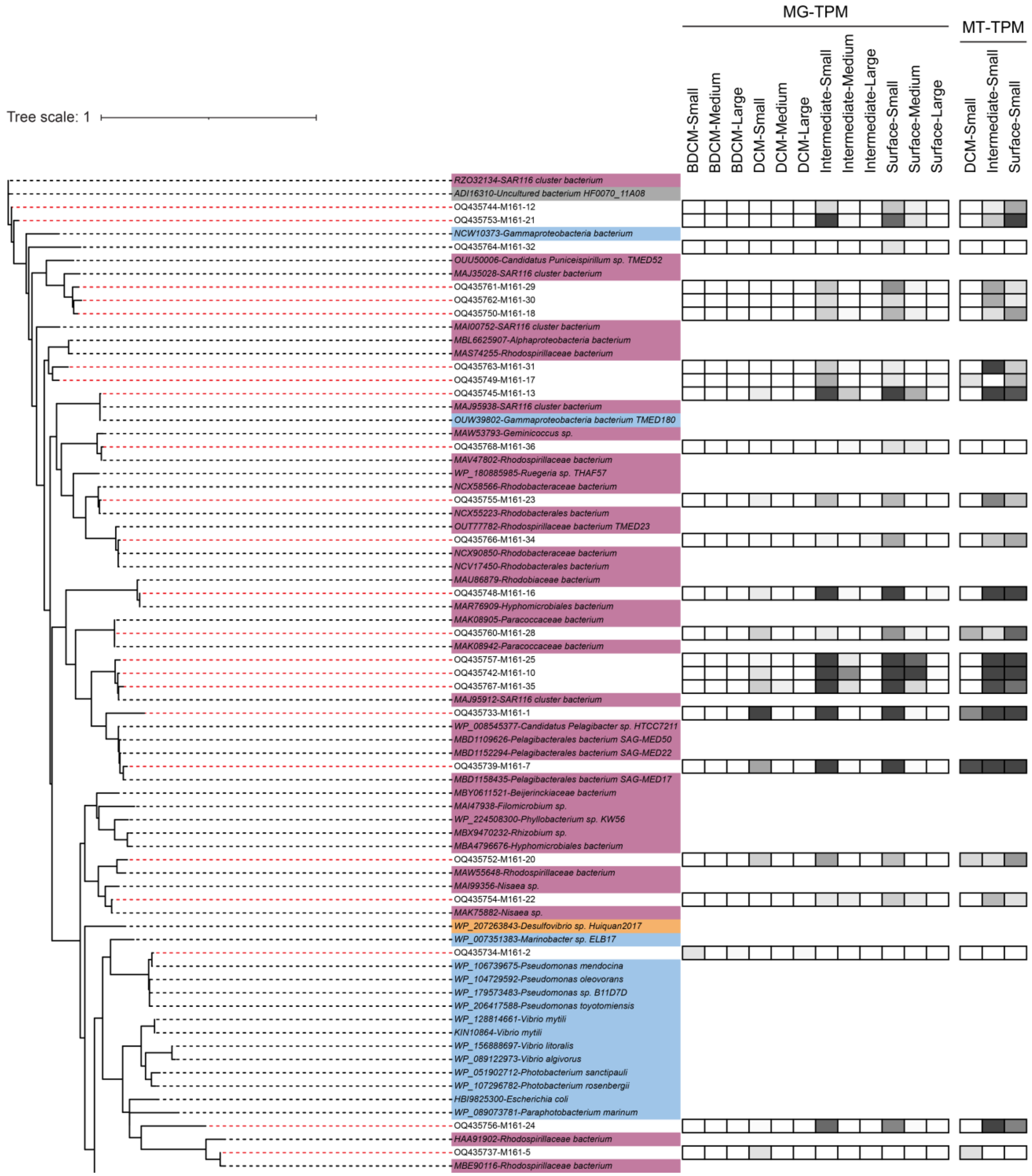

Figure S6. Continued

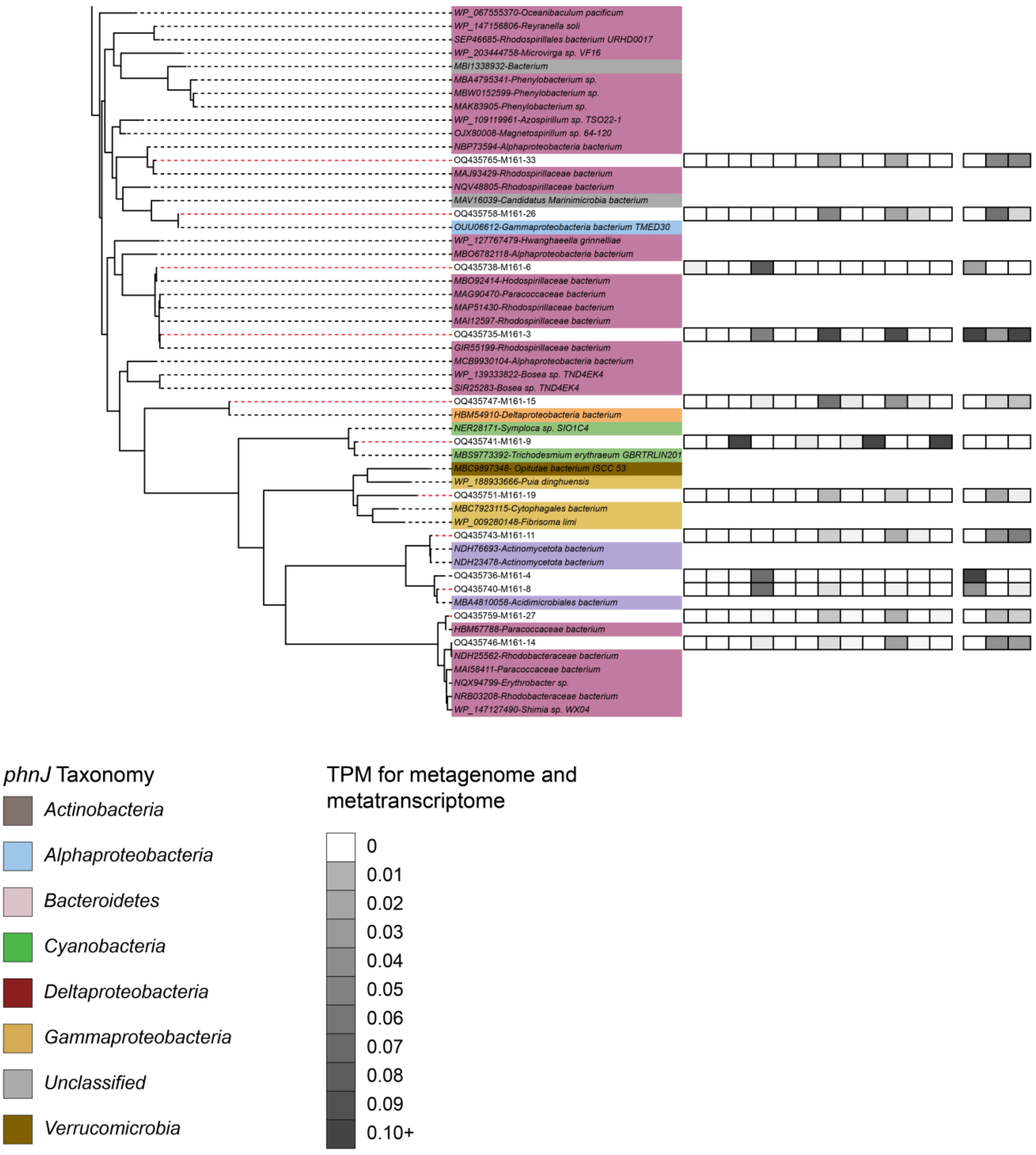

Figure S6. **Phylogenetic tree of the *phnJ* gene from Figure 4 with the reference sequences including accession numbers and TPM values.** All three size fractions: small (0.22 - 3 µm), medium (3 - 10 µm) and large (>10 µm) are included. The new sequences are indicated by the dashed red lines. Source data are provided as a source data file.

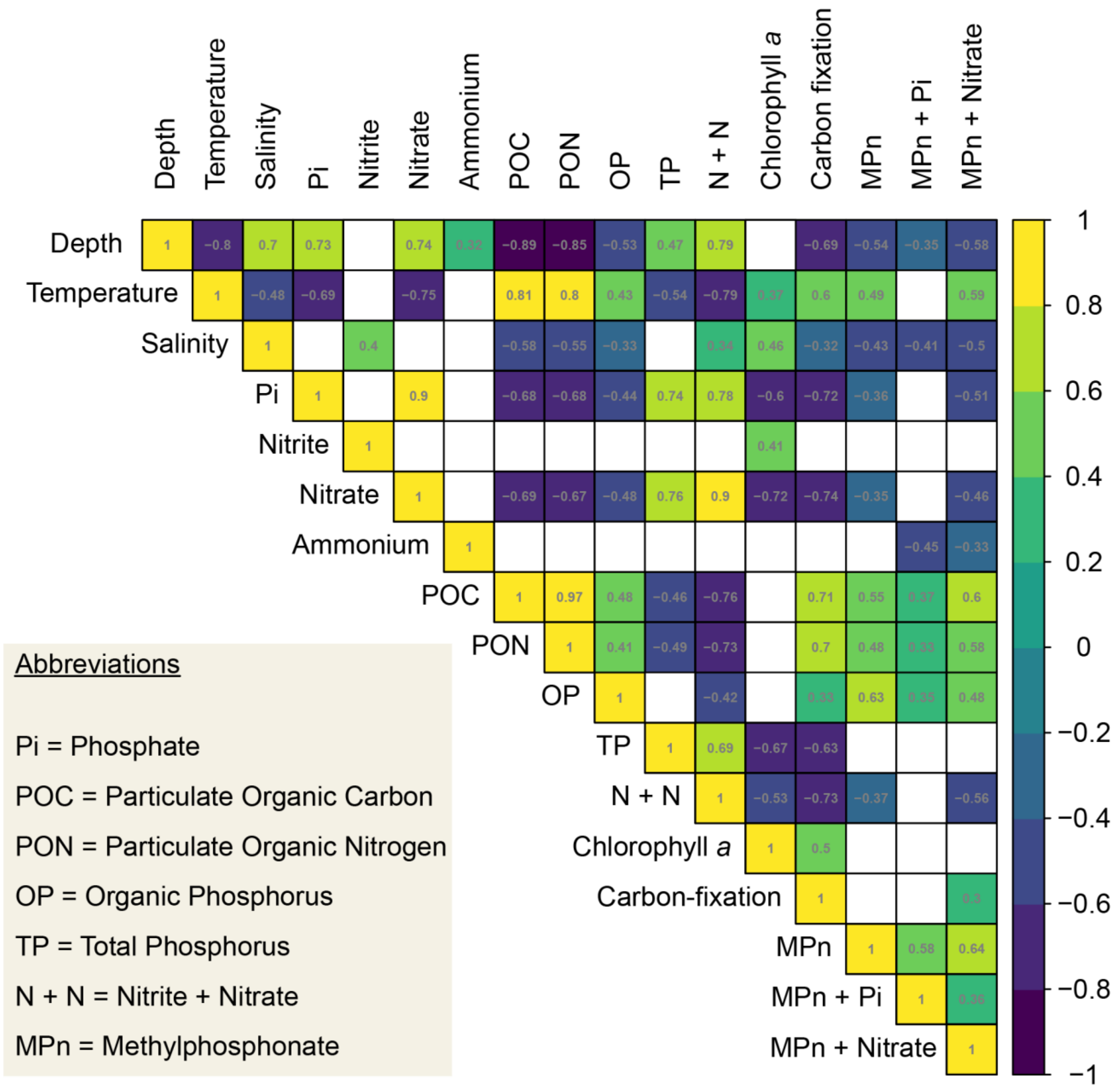

Figure S7. **Spearman ranked correlation analysis on all measured parameters.** All stations were used, except S2 where the nutrient data was unavailable. Insignificant correlations are left blank. Both mean carbon fixation and methylphosphonate-driven methane formation rates were used. Numbers in the squares indicate degree of correlation. Source data are provided as a source data file.

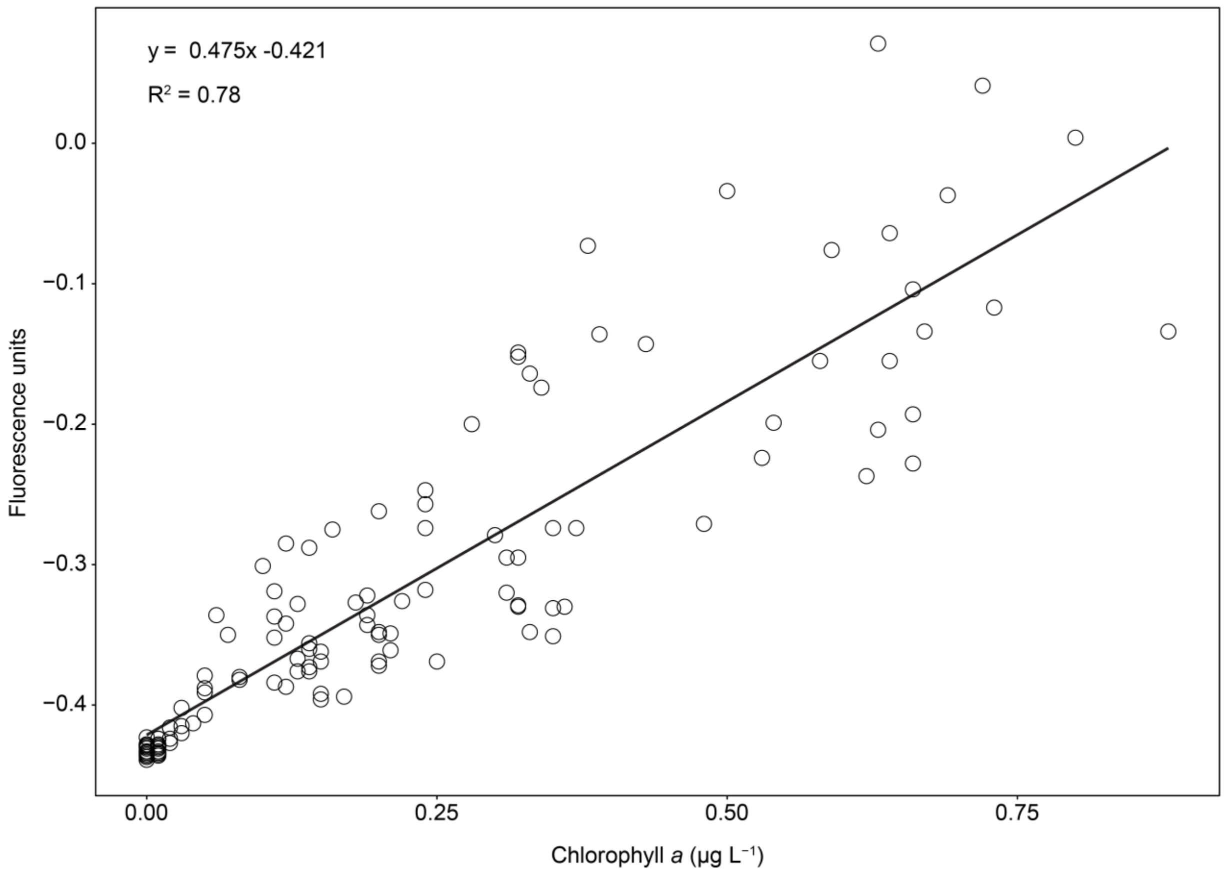

Figure S8. **Calibration of the fluorescence sensor with measured chlorophyll *a* (Chl *a*) values.** The limit of detection (calculated as the mean of the blanks plus three times the standard deviation) for the sensor (0.013 μg L^{-1}) was worse than for the measured Chl *a* values (0.008 μg L^{-1}). Therefore only the missing values (S3, S7, S1 201m, S8 201m and second CTD cast) were calibrated for plotting in Figure 1. Source data are provided as a source data file.

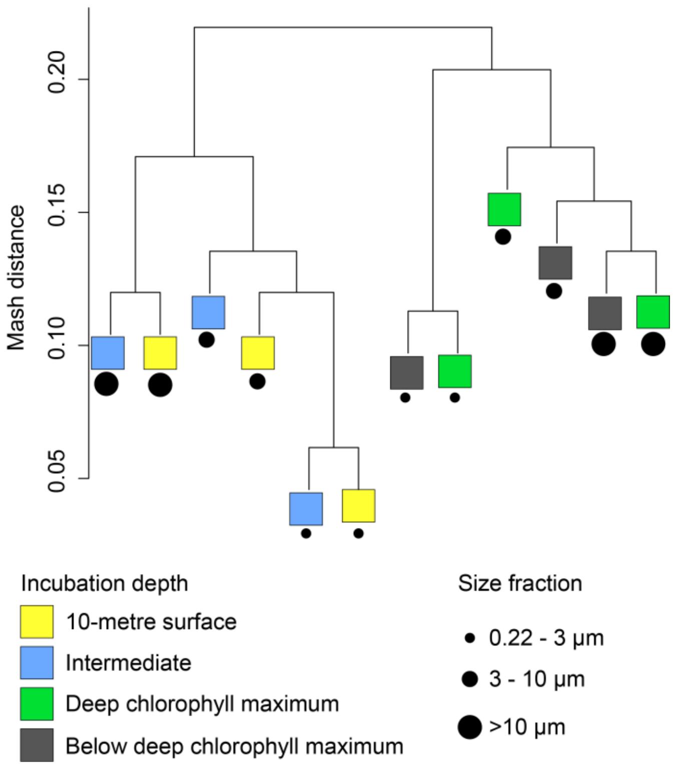

Figure S9. **Distance between the sequenced trimmed reads retrieved from station 5 as calculated by mash.** Two clear clusters formed between the two upper and two lower incubation depths. These are followed by subsequent clustering of the different size fractions. The 3 - 10 µm size fraction was clustering closer to the 0.22 - 3 µm one than the >10 µm fraction. As a result, samples were co-assembled in six co-assemblies based on size fraction and depth. Source data are provided as a source data file.

Supplementary Notes

Supplementary Note 1

Methylphosphonate-driven methane formation rate measurements

While only the data from the first 24 hours was used to quantify methylphosphonate-driven methane formation rates, our incubations lasted 48 hours, with one additional time point. Within this timeframe many incubations started to show exponential behaviour, a phenomenon we attribute to opportunistic growth under prolonged incubation times. This is especially expected due to the high ^{13}C-methylphosphonate supplementations as well as nutrient additions. Based on these observations, we caution against deriving rates of aerobic methane formation from incubations lasting over 24 hours, in which case multiple time points should be taken within the incubation time frame that allow for monitoring any potential onset of exponential rates.

In the methylphosphonate-supplemented incubations 22 out of 96 incubations did not show significant linear methane formation within the first 24 hours of incubation. However, all except one displayed substantial (28 - 4972 pmol L^{-1}) methane formation in the last time point, indicating that the capacity to metabolise methylphosphonate was there in most of the samples taken. Furthermore, 17 out of the 21 incubations were from the deep chlorophyll maximum (DCM) or the below DCM depths, suggesting that the methylphosphonate use is more variable in these depths. The single incubation without any formation was from the below DCM depth. In the experiment with equimolar amounts of phosphate (Pi) and methylphosphonate, 40 out of 90 incubations did not show significant linear methane formation within 24 hours of incubation, a clear increase from the methylphosphonate-supplemented experiment. However, methane formation was observed in 33 out of these incubations within 48 hours, suggesting that the capacity was still there, albeit at a reduced rate compared to the methylphosphonate-supplemented ones. The majority (25 out of 40) of incubations without linear rates were from the DCM and below DCM depths. In the methylphosphonate + nitrate-addition experiment 13 out of 91 incubations had no significant linear methane formation within 24 hours. Of these, only two had no quantifiable methane formation after 48 hours, originating from the DCM and below DCM depths. Overall, over prolonged incubation times methane formation can be quantified from most experiments but at a higher variability from the DCM and below DCM depths.

Supplementary Note 2

Abiotic photodegradation of methylphosphonate

It has been shown that methylphosphonate can decompose into methane and phosphate (Pi) under prolonged UV irradiation[2,3]. While we did not specifically test for photochemical degradation of methylphosphonate, we argue that the contribution of any such process is likely marginal at best and will not affect the rates measured in our study.

First of all, the conditions under which photochemical methylphosphonate degradation was shown to occur are not comparable to the conditions in our incubations or *in-situ*. Our incubation times were (much) shorter than the tested incubation times of 2100 hours[2] and 58 hours[3]. Furthermore, most efficient methylphosphonate degradation was observed under both highly alkaline and acidic conditions, unlike seawater which was expected to have a pH of around 8. Highest rates of abiotic methylphosphonate degradation were observed using a UV lamp with an intensity of 1200W[3], while our samples were incubated in natural light with Lee filter papers (Supplementary Figure S9).

Most importantly, our incubations (48 hours) covered two day and night cycles (with up to 12 h 15 minutes of darkness), whereas methane production proceeded linearly over time, without any clear relation to light availability (Supplementary Figure S4). Additionally, abiotic methane formation would be expected to produce similar amounts of methane in incubations exposed to the same light conditions and containing the same amount of ^{13}C-methylphosphonate tracer. This was not the case. Methane production in the surface and intermediate depths (which were incubated in the same incubator) varied by one to two orders of magnitude, as did even duplicate samples from the same depths. Additionally, water samples, to which phosphate was added had significantly lower methane production rates than the methylphosphonate incubations only, even though they were set up from the same water, with the same amount of methylphosphonate tracer and incubated under the same light conditions. We therefore conclude that the ^{13}C-methane produced in our incubations is a result of biological turnover of methylphosphonate and not photochemical degradation.

In-situ, the UV penetration depth Z10% for both UV-A and UV-B likely does not exceed 35 m ([4] and references therein) and thus would not significantly affect most incubation depths. Nonetheless, it would surely be worthwhile to test for photochemical methylphosphonate degradation under a range of environmentally-relevant settings (pH, irradiation intensity, temperature, time).

Supplementary Note 3

Methylphosphonate utilisation in the presence of phosphate

Methane production in the presence of Pi might be an indication for a persistent expression of the C-P lyase pathway. It is conceivable that competition for methylphosphonate or phosphonates in general is less fierce than for inorganic phosphate, hence their utilisation might offer an ecological advantage, particularly if carbon is limiting. Alternatively, some organisms may be able to afford to express pathways that allow them to co-metabolise organic phosphorus compounds (such as methylphosphonate) and inorganic phosphate, as shown for *Trichodesmium*[5]. Additionally, the enzyme might be expressed to e.g. deal with the degradation of phosphonates, which might have cytotoxic or protective effects, rather than for the purpose of obtaining phosphorus[6,7]. It would be of great interest to explore whether there is a universal threshold inhibitory Pi concentration that turns methylphosphonate utilisation on and off or whether this value responds to the respective biogeochemical parameters (e.g. availability of carbon, nutrients and dominant methylphosphonate utilising taxa) of a given environment.

Supplementary Note 4

Trichodesmium as a phosphonate producer and consumer

Trichodesmium - together with *SAR11*[8,9] - belongs to the small group of organisms that have the capacity to be both phosphonate producers and consumers[7]. The capacity to degrade phosphonates with the C-P lyase pathway seems to be widespread in the *Trichodesmium* genus and includes *T. erythraeum*, *T. thiebautii*, *T. tenue*, and *T. spiralis*[10] as well as members of its microbiome[11]. On the other hand, the synthesis of phosphonates has only been confirmed for *T. erythraeum*[12] as well as the microbiome[11]. It has been proposed that phosphonate formation and consumption may additionally be strain specific[7,13].

Phosphonate synthesis may be temporally or spatially separated from phosphonate utilisation, for example during the vertical migration in the water column[14] between Pi rich bottom waters and Pi deplete surface waters, which may promote phosphonate synthesis (the former) or utilisation (the latter). Additionally, an organism like *Trichodesmium*, which has a huge genome (7.75 Mbp[15]) and lives under energy surplus may afford to encode and express ‘luxury’ metabolic pathways that are not key for its survival. It has also been proposed that *Trichodesmium* may produce phosphonates to restrict access

to this critical nutrient to microbes other than its own epibionts[16]. Phosphonate synthesis and degradation might also serve other purposes than phosphorus acquisition, such as defence, as many phosphonates are potent antibiotics[7]. In any case, the capacity to both produce and utilise organic phosphorus compounds may decisively contribute to the ecological success of *Trichodesmium* in low phosphate, oligotrophic systems[10].

Supplementary Note 5

Origin of methylphosphonate in surface waters remains elusive

While the carbon phosphorus (C-P) lyase pathway is widespread in the surface waters, the origin of methylphosphonate (and phosphonates in general) in these waters is still largely unresolved. The genetic potential for methylphosphonate synthesis is inferred based on the presence of the *mpnS* gene encoding for methylphosphonate synthase[17]. In our samples, we found only a few fragmented *mpnS* gene-related sequences (data not shown), predominantly from below the DCM and associated with the ammonia oxidising *Thaumarchaeota*. Indeed, the ammonia-oxidising *Nitrosopumilus maritimus*[17] is - together with *Pelagibacter ubique SAR11*[9] - the only organism with a confirmed capacity to synthesise methylphosphonate. However, there are indications that other pro- and eukaryotes may have the capability to form methylphosphonate as well[9]. For example, abundant cyanobacteria like *Proclorococcus* and *Trichodesmium* have the capacity to synthesise phosphonates[7,12]. However, so far neither *Prochlorococcus*, *Trichodesmium* or its associated microbiome has been shown to have the capacity to synthesise methylphosphonate specifically[16]. Both *N. maritimus* and *P. ubique SAR11* are some of the most abundant microorganisms in the marine environment[18,19]. While *SAR11* can be abundant in the euphotic surface ocean, *N. maritimus* is usually found below the photic layer. This conforms to the observation that phosphonate synthesis generally increases below 100 m depth[7], presumably due to the increased availability of Pi required for phosphonate synthesis. This is further supported by a global bioinformatic analysis that looked the *mpnS* more specifically in conjunction with other phosphonate genes[20]. They found that the *mpnS* was present in 7.7% of the community inhabiting the mesopelagic ocean and in less than 1.5% of those inhabiting the surface and DCM but their transcripts remained below 0.25 per 100000 reads. The supply of phosphonates to the surface waters must then be achieved by means of physical mixing across the thermocline[21]. However, this transport, like that of Pi, is probably restricted and it thus remains an open question whether there are additional biological sources of methylphosphonate in marine surface waters.

References

1. Duerschlag, J. *et al.* Niche partitioning by photosynthetic plankton as a driver of CO2-fixation across the oligotrophic South Pacific Subtropical Ocean. *ISME J.* **16**, 465–476 (2022).

2. Yu, C., Wang, F., Chang, S. J., Yao, J. & Blake, R. E. Phosphate oxygen isotope evidence for methylphosphonate sources of methane and dissolved inorganic phosphate. *Sci. Total Environ.* **644**, 747–753 (2018).

3. Zhang, C. & Ji, H. B. Effects of environmental parameters on the ultraviolet degradation of methylphosphonate. *Appl. Ecol. Environ. Res.* **17**, 9473–9482 (2019).

4. Tedetti, M. & Sempéré, R. Penetration of Ultraviolet Radiation in the Marine Environment. A Review. *Photochem. Photobiol.* **82**, 389 (2006).

5. Beversdorf, L. J., White, A. E., Björkman, K. M., Letelier, R. M. & Karl, D. M. Phosphonate metabolism by Trichodesmium IMS101 and the production of greenhouse gases. *Limnol. Oceanogr.* **55**, 1768–1778 (2010).

6. Horsman, G. P. & Zechel, D. L. Phosphonate Biochemistry. *Chem. Rev.* **117**, 5704–5783 (2017).

7. Acker, M. *et al.* Phosphonate production by marine microbes: Exploring new sources and potential function. *Proc. Natl. Acad. Sci. U. S. A.* **119**, (2022).

8. Carini, P., White, A. E., Campbell, E. O. & Giovannoni, S. J. Methane production by phosphate-starved SAR11 chemoheterotrophic marine bacteria. *Nat. Commun.* **5**, 1–7 (2014).

9. Born, D. A. *et al.* Structural basis for methylphosphonate biosynthesis. *Science (80-.).* **358**, 1336–1339 (2017).

10. Dyhrman, S. T. *et al.* Phosphonate utilization by the globally important marine diazotroph Trichodesmium. *Nature* **439**, 68–71 (2006).

11. Frischkorn, K. R., Rouco, M., Van Mooy, B. A. S. & Dyhrman, S. T. Epibionts dominate metabolic functional potential of Trichodesmium colonies from the oligotrophic ocean. *ISME J.* **11**, 2090–2101 (2017).

12. Dyhrman, S. T., Benitez-Nelson, C. R., Orchard, E. D., Haley, S. T. & Pellechia, P. J. A microbial source of phosphonates in oligotrophic marine systems. *Nat. Geosci.* **2**, 696–699 (2009).

13. Schowanek, D. & Verstraete, W. Phosphonate utilization by bacterial cultures and enrichments from environmental samples. *Appl. Environ. Microbiol.* **56**, 895–903 (1990).

14. Villareal, T. A. & Carpenter, E. J. Buoyancy regulation and the potential for vertical migration in the oceanic cyanobacterium Trichodesmium. *Microb. Ecol.* **45**, 1–10 (2003).

15. Bergman, B., Sandh, G., Lin, S., Larsson, J. & Carpenter, E. J. Trichodesmium - a widespread marine cyanobacterium with unusual nitrogen fixation properties. *FEMS Microbiol. Rev.* **37**, 286–302 (2013).

16. Frischkorn, K. R. *et al.* Trichodesmium physiological ecology and phosphate reduction in the western tropical South Pacific. *Biogeosciences* **15**, 5761–5778 (2018).

17. Metcalf, W. W. *et al.* Synthesis of Methylphosphonic Acid by Marine Microbes: A Source for Methane in the Aerobic Ocean. *Science (80-.).* **337**, 1104–1107 (2012).

18. Giovannoni, S. J. SAR11 Bacteria: The Most Abundant Plankton in the Oceans. *Ann. Rev. Mar. Sci.* **9**, 231–255 (2017).

19. Karner, M. B., Delong, E. F. & Karl, D. M. Archaeal dominance in the mesopelagic zone of the Pacific Ocean. *Nature* **409**, 507–510 (2001).

20. Lockwood, S., Greening, C., Baltar, F. & Morales, S. E. Global and seasonal variation of marine phosphonate metabolism. *ISME J.* **16**, 2198–2212 (2022).

21. Cavender-Bares, K. K., Karl, D. M. & Chisholm, S. W. Nutrient gradients in the western North Atlantic Ocean: Relationship to microbial community structure and comparison to patterns in the Pacific Ocean. *Deep. Res. Part I Oceanogr. Res. Pap.* **48**, 2373–2395 (2001).
